# Interstitial lung abnormalities: Real‐world evidence

**DOI:** 10.1002/hsr2.1216

**Published:** 2023-04-21

**Authors:** Carina Rôlo Silvestre, Catarina Custódio, Susana Clemente, Silvia Rodrigues, Sofia Tello Furtado, Rui Maio

**Affiliations:** ^1^ Pulmonology Department Centro Hospitalar do Oeste Torres Vedras Portugal; ^2^ Hospital Beatriz Ângelo Loures Portugal

**Keywords:** Interstitial lung abnormalities, Interstitial lung disease, Enhanced Recovery After Surgery program

To the Editor,

Interstitial lung abnormalities (ILAs) are a radiological entity characterized by incidental findings in chest computed tomography (CT) scans, with specific patterns of modifications in lung density in patients with no prior history of interstitial lung disease. These radiologic changes are present in more than 5% of nondependent lung parenchyma areas. And they include reticular abnormalities or ground‐glass attenuation, lung distortion, traction bronchiectasis/bronchiolectasis, honeycombing, and nonemphysematous cysts.^1^, ^2^ In some cases, ILA may represent an early form of pulmonary fibrosis.^1^, ^2^, ^3^

ILA affects about 7% of the general population. The prevalence varies with age and with smoking habits, being more prevalent in older and smoker individuals.^1^, ^3^, ^4^ These patients present more respiratory symptoms and changes in respiratory function tests, particularly with a decrease in forced vital capacity.^5^, ^6^, ^7^ This entity seems related to raised proinflammatory molecules that lead to radiological changes.^4^ This radiological concept has clinical implications and higher morbidity and mortality.^4^, ^5^, ^7^, ^8^, ^9^ The recognition of its clinical importance has been increasing in recent years. Identifying ILA patients with progressive disease or at risk of dying is of utmost clinical importance.

Therefore, we designed a study to identify and characterize patients with ILA within the Enhanced Recovery After Surgery (ERAS®) program.

We conducted a retrospective and descriptive study of patients referred to Pulmonary Rehabilitation in the preoperative period of major abdominal surgery, within the scope of the ERAS® program. We included patients with benign diseases and cancer, regardless of disease stage. All surgeries were programmed. The data was collected by analyzing clinical processes. We analyzed 21 months between January 2020 and October 2021.

Statistical analysis was performed using SPSS software (version 23.0). Continuous variables were presented as mean ± standard deviation or median and respective interquartile range (IQR), depending on the normality of the underlying data. Categorical variables were compared with the *χ* ^2^ test. The significance value was taken as *p* < 0.05.

We considered a period until 60 days after hospital discharge to analyze mortality.

We evaluated 128 patients with a median age of 71 years (IQR: 56–86), most of them were male (*n* = 82; 64%), and 50.8% (*n* = 65) had a history of smoking (14.8% were current smokers and 35.9% ex‐smokers). Patients presented a median body mass index of 26.1 kg/m^2^ ± SD 5.031.

Most patients were referred to the ERAS® program for oncological disease (*n* = 122; 95.3%) with curative intent, mainly of the gastrointestinal tract (*n* = 71; 55.5%), and had done a chest CT scan for staging their disease.

In this study, we found seven (5.5%) patients having ILA. Most of them were male (*n* = 6; 85.7%), with a median age of 78 years (IQR: 49–107).

About 42.9% (*n* = 3) of ILA patients had a smoking history. Two patients had respiratory symptoms, and none of those who underwent functional assessment had alterations (*n* = 5). The radiologic evaluation revealed that most patients (*n* = 6) had reticular alterations.

ILA patients had more comorbidities than non‐ILA patients (*p* = 0.033). We found an association between gastroesophageal reflux disease (GERD) and the presence of ILA (odds ratio [OR]: 15.73; 95% confidence interval [CI]: 2.128–116.314; *p* = 0.024) (Table 1).

**Table 1:** Association between the presence of comorbidities and ILA.

Comorbidities	*p* Value
GERD	0.024
Auricular fibrillation	0.513
Diabetes	0.090
Pulmonary embolism	0.546
Hypertension	0.576
Cardiac insufficiency	0.583
Ischemic heart disease	0.366

Abbreviations: GERD, gastroesophageal reflux disease; ILA, interstitial lung abnormalities.

Other studies point out that patients with ILA are more likely to be diagnosed with respiratory, malignant, or cardiovascular diseases compared with those without ILA.^7^

The median length of hospital stay was 9 days (IQR: 7–24) in ILA versus 7 days (IQR: 5–14) in non‐ILA patients (*p* = 0.205).

Seven patients died, of which three had ILA. We found an association between having ILA and death (OR: 17.4; 95% CI: 3.041–99.552; *p* = 0.005).

This study has some limitations, namely the modest sample size and its observational design. Additionally, there was no specific protocol for performing CT scans and identifying ILA. Nevertheless, these patients were assessed by one pulmonologist, who used the same ILA criteria for all patients.

In conclusion, this study describes the real‐world evidence of ILA in major abdominal surgery. It shows that of the patients submitted to the ERAS® program, 5.5% had ILA, corresponding to the prevalence described in other studies. We also found that these patients have more associated pathologies than patients without ILA. From the analysis of comorbidities, as identified in other studies, GERD is associated with ILA.

ILA patients have a higher risk of mortality. Thus, identifying ILA patients has essential prognostic value. It is crucial to design prospective studies with a significant number of patients to determine the natural history of ILA.

## AUTHOR CONTRIBUTIONS

**Carina Rôlo Silvestre**: Conceptualization; data curation; formal analysis; investigation; writing—original draft. **Catarina Custódio**: Data curation; investigation. **Susana Clemente**: Conceptualization; formal analysis; investigation; methodology; supervision; writing—review and editing. **Silvia Rodrigues**: Data curation. **Sofia Tello Furtado**: Formal analysis; validation; writing—review and editing. **Rui Maio**: Validation; writing—review and editing.

## CONFLICT OF INTEREST STATEMENT

The authors declare no conflict of interest.

## ETHICS STATEMENT

The study was approved by the Ethics Committee.

## TRANSPARENCY STATEMENT

The lead author Carina Rôlo Silvestre affirms that this manuscript is an honest, accurate, and transparent account of the study being reported; that no important aspects of the study have been omitted; and that any discrepancies from the study as planned (and, if relevant, registered) have been explained.

## Data Availability

All data are available upon request to the author.
